# Comparative Toxigenicity and Associated Mutagenicity of *Aspergillus fumigatus* and *Aspergillus flavus* Group Isolates Collected from the Agricultural Environment

**DOI:** 10.3390/toxins12070458

**Published:** 2020-07-17

**Authors:** Caroline Lanier, David Garon, Natacha Heutte, Valérie Kientz, Véronique André

**Affiliations:** 1 Faculty of Health, Normandie University, UNICAEN, Centre F. Baclesse, UR ABTE EA4651, 14000 Caen, France; caroline.lanier@univ-lille.fr (C.L.); david.garon@unicaen.fr (D.G.); 2 Faculty of Sports, Normandie University, UNIROUEN, CETAPS EA3832, 76821 Mont Saint Aignan CEDEX, France; natacha.heutte@univ-rouen.fr; 3 Laboratoire LABEO, Route de Rosel, 14280 Saint-Contest, France; valerie.kientz@calvados.fr

**Keywords:** toxigenicity, mutagenicity, mycotoxin mixtures, mycotoxin interactions, Aspergilli

## Abstract

The mutagenic patterns of *A. flavus, A. parasiticus* and *A. fumigatus* extracts were evaluated. These strains of toxigenic *Aspergillus* were collected from the agricultural environment. The Ames test was performed on *Salmonella typhimurium* strains TA98, TA100 and TA102, without and with S9mix (exogenous metabolic activation system). These data were compared with the mutagenicity of the corresponding pure mycotoxins tested alone or in reconstituted mixtures with equivalent concentrations, in order to investigate the potential interactions between these molecules and/or other natural metabolites. At least 3 mechanisms are involved in the mutagenic response of these aflatoxins: firstly, the formation of AFB_1_-8,9-epoxide upon addition of S9mix, secondly the likely formation of oxidative damage as indicated by significant responses in TA102, and thirdly, a direct mutagenicity observed for higher doses of some extracts or associated mycotoxins, which does not therefore involve exogenously activated intermediates. Besides the identified mycotoxins (AFB_1_, AFB_2_ and AFM_1_), additional “natural” compounds contribute to the global mutagenicity of the extracts. On the other hand, AFB_2_ and AFM_1_ modulate negatively the mutagenicity of AFB_1_ when mixed in binary or tertiary mixtures. Thus, the evaluation of the mutagenicity of “natural” mixtures is an integrated parameter that better reflects the potential impact of exposure to toxigenic Aspergilli.

## 1. Introduction

We have previously demonstrated in an agricultural environment (Normandy, France), that oilseed cakes and maize silage used for cattle food could be contaminated with Aspergilli, especially *A. flavus, A. parasiticus* and *A. fumigatus*, even in temperate/mild climate [1,2]. Moreover, bioaerosols resulting from breeding activities were also contaminated by airborne molds [3,4]. Among them, some species belonging to *Aspergillus* genus are well-known as potential producers of various mycotoxins such as aflatoxins, gliotoxin, verruculogen or fumagillin. The toxic effects of these purified mycotoxins (carcinogenicity, cytotoxicity, neurotoxicity, immunosuppressive effects) have been widely described so far [5,6,7,8,9]. Thus, aflatoxin B_1_ (AFB_1_), produced by the *Aspergilli flavus* group, had well known mutagenic and genotoxic properties and is classified in group 1 by the International Agency for Research on Cancer (IARC), due to its carcinogenic potential [10]. Gliotoxin, produced by *A. fumigatus*, was found to be genotoxic using the bacterial repair assay (*E. coli* WP2 strain vs. its repair deficient derivative CM871) and the comet assay on RAW264.7 macrophages (cell line issued from mice). In contrast, no significant effect was reported in the *Salmonella* test nor in the SOS-chromotest or in Chinese hamster ovarian (CHO) cells for the induction of sister chromatide exchanges (SCE) [11]. Stanimirovic et al. [12] and Stevanovic et al. [13] suggested that fumagillin had clastogenic and cytotoxic potentials (inducing micronuclei (MN) and chromosomal aberrations (CA)) in vitro (human lymphocytes) and in vivo (mice BALB/c). Verruculogen, also produced by *A. fumigatus*, was reported as mutagenic towards various strains in the *Salmonella*/mammalian-microsome assay (Ames test) [14]. In contrast, little is known about the mutagenic properties of the mixtures of secondary metabolites naturally produced by environmental fungal isolates. Bjeldanes et al. [15] reported that chloroformic extracts obtained from cultures of *A. flavus* and *A. parasiticus* displayed a mutagenic behaviour in TA100 in the presence of S9mix (Ames test).

In this context, the aim of the present study was to characterize the mutagenic patterns of *A. flavus, A. parasiticus* and *A. fumigatus* extracts, prepared from cultures of fungal strains previously isolated from the occupational agricultural environment. Comparisons between mycotoxin-producer and non-producer isolates were performed using the Ames test on strains TA98, TA100 and TA102, without and with S9mix addition. These data were then compared with the mutagenicity of the corresponding pure mycotoxin standards tested alone or in reconstituted binary and ternary mixtures with equivalent concentrations, in order to examine the potential interactions between these molecules and the involvement of other natural metabolites.

## 2. Results

### 2.1. Mycotoxin Production

Toxigenic *A. flavus* and *A. parasiticus* strains both synthesize AFB_1_ and, to a lesser extent, AFB_2_, whereas AFM_1_ is naturally produced only by the toxigenic *A. flavus* (Table 1). Mycotoxin production is 20- to 40-fold higher for *A. flavus* as compared to *A. parasiticus*. None of these mycotoxins were detected for the so-called “non toxigenic” corresponding strains. The two strains of *A. fumigatus* were toxigenic, producing roughly comparable fumagillin and verruculogen levels, but they differ with regard to their gliotoxin synthesis potential (Table 1).

### 2.2. Mutagenicity of Fungal Extracts

Mutagenicity was evaluated using the Ames test in three *Salmonella* tester strains with and without exogenous metabolic activation system (S9mix). TA98 is sensitive to chemicals acting through frameshift mechanism, whereas TA100 and TA102 are sensitive to chemicals acting through base pair substitutions mechanism.

#### 2.2.1. *Aspergillus flavus* Extracts

The mutagenic potential of the (Flav^+^) extract is displayed in Table 2 (direct mutagenicity) and Table 3 (upon S9mix addition). Significant responses were observed in the three *Salmonella* tester strains, in absence as well as in presence of S9mix, except for strain TA102 for which no direct mutagenicity was observed.

Moreover, significant mutagenicities were systematically coupled with a clear dose–response relationship, and were dramatically increased in the presence of S9mix (Table 3). In contrast, no mutagenic response was observed from the (Flav−) extract.

#### 2.2.2. *Aspergillus parasiticus* Extract

No mutagenic activity was detected for the (Para^−^) extract (Table 4 and Table 5). In contrast, upon S9mix addition, the (Para^+^) extract was mutagenic in the three *Salmonella* tester strains (Table 5), with a dose-response relationship. Without S9mix (Table 6), a dose-dependent mutagenic response was obtained in TA98 only.

#### 2.2.3. *Aspergillus fumigatus* Extract

There was no mutagenic activity observable in *Salmonella* tester strains TA98 and TA100 (Table 6 and Table 7). In TA102, both extracts presented a low mutagenicity only upon addition of S9mix (Table 7).

### 2.3. Mutagenicity of the Mycotoxin Mixtures

Reconstituted mixtures were prepared from pure mycotoxins with concentrations corresponding to those measured in extracts. These binary and ternary mixtures were then tested for their mutagenic potential.

Compared to their corresponding extracts (Flav^+^) and (Para^+^) (Table 2, Table 3, Table 4 and Table 5), the respective mixtures prepared from pure aflatoxins were systematically and significantly less mutagenic (2 to 10-fold; p < 10^−4^) towards the three *Salmonella* tester strains. In the range of concentrations tested, the binary mixture (AFB_2_ + AFM_1_) was not mutagenic (Table 2 and Table 3), nor were the mixtures including (fumagillin + verruculogen ± gliotoxin), in the three *Salmonella* strains used (Table 6 and Table 7).

### 2.4. Mutagenicity of Pure Mycotoxins

Mutagenic activities of the 6 mycotoxins detected in the different extracts (Table 1) were also evaluated from pure standard solutions (Table 8). In a range of concentrations equivalent (or even wider) to those tested with the whole extracts, AFB_1_ was the only one displaying a significant mutagenicity in TA98 and TA100, both without and with S9mix. In contrast, no mutagenic response was observed in TA102.

## 3. Discussion

Various Aspergilli species were isolated from our previous studies conducted in an agricultural environment. They differed considering both their ability to synthesize mycotoxins and the nature of these mycotoxins (Table 1). They originated from contaminated oilseed cakes or from bioaerosols sampled in dairy cattle sheds or during cattle feed distribution. Thus, spores and mycelium fragments of these Aspergilli species could be ingested by livestock and also inhaled by farmers during their daily work. In order to evaluate the potential hazard associated with occupational exposure to these fungal strains, we measured the mutagenic potential of total extracts obtained from MEA cultures. For this purpose, *Salmonella typhimurium* TA98, TA100 and TA102 tester strains were used (Ames test) without and with the exogenous metabolic fraction S9mix. The mutagenicity of the pure mycotoxins at the same concentrations as in the extracts was evaluated and compared in order to investigate the presence of additional mutagenic compounds in these extracts. It is noteworthy that, in the range of concentrations studied, corresponding to native production levels, AFB_1_ was the sole mycotoxin displaying a significant mutagenicity when tested alone. Thus, extracts mutagenicity was discussed in comparison with that of an equivalent pure AFB_1_ concentration (Table 9).

AFB_1_ and AFB_2_ were found in the (Flav^+^) extract and, to a lesser extent, in the (Para^+^). It must be stressed that AFM_1_, which is mainly considered as a liver-hydroxylated metabolite of AFB_1_, was detected in the (Flav^+^) extract. This direct production of AFM_1_ by *A. flavus* was also recently reported by Uka et al. [16], but does not appear to have been commonly described.

A significant direct mutagenicity was observed in TA98 for (Flav^+^) and (Para^+^) extracts as well as for pure AFB_1_. Previously, Loarca-Piña et al. [17] reported a direct mutagenicity for AFB_1_, using the microsuspension assay. Compared with our study, they reported comparable mutagenic potencies for TA100 (0.5 vs. 0.54 revertants/ng) but somewhat higher for TA98 (0.4 *vs* 0.18 revertants/ng). In the present work, the responses observed for (Flav^+^) and even more for (Para^+^) extracts were largely enhanced (2- to 8-fold) compared with the same dose of pure AFB_1_ (Table 9). Similarly, a higher direct mutagenicity was obtained in TA100 only for the (Flav^+^) extract, compared to pure AFB_1_ (Table 9).

Upon addition of S9mix, the mutagenic activities dramatically increased (50- to 100-fold) in both strains TA100 and TA98 (Table 9). Under these conditions, (Flav^+^) and (Para^+^) extracts are 2 to 4-fold more mutagenic than AFB_1_ alone. The (Para^+^) extract was a more potent mutagen than (Flav^+^) in TA98 (71.6 vs. 47.3 revertants/ng AFB_1_), whereas in TA100, (Flav^+^) appeared the most mutagenic (172 vs. 99 revertants/ng AFB_1_). Additionally, in TA102, comparable significant responses were obtained for (Flav^+^) and (Para^+^) extract (5.5 and 4.4 revertants/ng AFB_1_ respectively), but not for pure AFB_1_.

Thus, these patterns indicate that AFB_1_ is only one of the direct- and indirect-mutagenic compounds present in the total extracts. Moreover, the absence of mutagenicity for (Flav^-^) and (Para^-^) extracts could indicate that additional metabolites are closely related to the AFB_1_ synthesis pathway. Recently, Uka et al. [16] highlighted the particularly high intra-species diversity of *A. flavus* and reported the large variety of secondary metabolites that can be produced. Using a metabolomic approach on 55 isolates of *A. flavus*, they identified more than 50 metabolites even if only half of the 55 strains were considered as aflatoxin producers. Beside aflatoxins (AFB_1_, AFB_2_ and possibly AFB_2a_, AFM_1_, AFG_1_, AFG_2_) and their known precursors (sterigmatocystin and derived compounds), biocoumarins (aflavarin and derived compounds) with anti-insectan activity, isocoumarines (asperentin and derived compounds), anthraquinones (asparasone and derived compounds), non-ribosomal peptides (aspergillic acid and derived compounds) with antibiotic activity and indol-diterpenoids (aflavinines and aflatrem) with tremorgenic potential were identified. However, among these metabolites, only sterigmatocystin was reported as mutagenic in Ames test, both in TA100 (400 revertants/0.1 µg with S9mix and 150 revertants/0.1 µg without S9mix) and in TA98 (190 revertants/0.1 µg with S9mix and 40 revertants/0.1 µg without S9mix) [18].

Aflatoxicol displayed also a mutagenic potential in the presence of mammalian microsomes [19]. The production of aflatoxicol by *A. flavus*, as well as its possible interconversion with AFB_1_ have been previously reported by Nakazato et al. [20,21].

Moreover, the mutagenic activities measured from (Flav^+^) and (Para^+^) extracts were always significantly higher than those obtained with the corresponding reconstituted mixtures of pure mycotoxins. These observations argue again in favour of additional components extracted from fungal cultures, and contributing to the overall mutagenic response.

The potential interactions between identified mycotoxins were also considered. Thus, the mutagenicity of reconstituted mixtures was compared to that of pure mycotoxins tested individually. Despite the fact that AFB_2_ and AFM_1_ were not mutagenic per se in our conditions, their association with AFB_1_ was systematically linked to a significantly decreased mutagenicity of the corresponding binary or ternary mixtures, indicating a potential negative interaction between these structurally close molecules. A competitive mechanism during the S9 metabolic activation step could be evoked. Since AFB_2_ and AFM_1_ are structurally close to AFB_1_, they could also be substrate for cytochrome P450 enzymes (CYP), leading to a competition between these mycotoxins for the binding step to enzyme. Interaction could also occur later, once the 8,9-epoxide metabolites formed. Indeed, a transient intercalation step precedes the formation of DNA adducts [22,23]. This intercalation in the 5′ side of the guanine target facilitates further adduct formation by favourably positioning the epoxide for subsequent nucleophilic attack by the guanine N7 [23,24]. The metabolites derived from AFB_2_ and AFM_1_ could potentially interact with this transient intercalation state, resulting in a decreased mutagenicity of AFB_1_ tested in binary or tertiary mixtures. But, since the negative modulation on mutagenicity was also observed without S9mix, additional but as yet unidentified interaction targets must be involved, independently of the metabolic activation step.

Indeed, at least three mechanisms are presumably involved in the mutagenic responses of these aflatoxins:

(a) the most powerful and classically described towards TA98 and TA100 upon addition of S9mix involves CYPs in the formation of reactive intermediates such as AFB_1_-*exo*-8,9-epoxide, and the subsequent binding to the N7 guanine in DNA to form AFB_1_-N7-Gua adducts then, subsequently, the imidazole ring-opened form AFB_1_-FapyGua, this latter being more mutagenic than the former [25,26];
(b) a direct mutagenicity towards these two strains, observed for higher doses of mycotoxins, that did not involve any exogenously activated intermediates;
(c) and a mechanism detected in TA102 for the three extracts only upon addition of S9mix, could potentially be subsequent to oxidative damage, since TA102 is responsive to this kind of damage [27].

It has been reported that exposure to AFB_1_ could led to oxidative damage, either in vitro [28,29,30,31] or in vivo [9,31,32,33]. Hence, Guindon et al. [34] reported an elevation of 8-hydroxy-2′-deoxyguanosine (8-OH-dG) adducts in mice lung, and more recently, Coskun et al. [35] described an optimized protocol to detect (5′R)- and (5′S)-8,5′-cyclo-2′-deoxyadenosines in mice liver. Thus, it is now currently recognized that this mechanism contributes to the overall toxicity and genotoxicity of this mycotoxin. However, pure AFB_1_ was never reported mutagenic in TA102, thus the mutagenicty observed with extracts should be attributed to other secondary metabolites.

Verruculogen and fumagillin were produced in the same order of magnitude by the two strains of *A. fumigatus* that differed regarding their capacity to synthesize gliotoxin. These extracts did not display any direct mutagenicity, but borderline responses were obtained for both extracts in TA102 upon addition of S9mix. When purified standards were tested in parallel, none of them were found mutagenic in the range of concentrations corresponding to the native production of *A. fumigatus* strains. These data are in accordance with previous results for gliotoxin [11,36]. Even if verruculogen was reported as mutagenic in TA98 and TA100 [14], the tested doses were about four orders of magnitude higher than those tested in the present study. Fumagillin was reported clastogenic, inducing AC and MN [12], but no data were found regarding the mutagenic potency in the *Salmonella* assay. Thus, as previously suggested for (Flav^+^) and (Para^+^) extracts, identified mycotoxins or their interactions cannot explain the weak mutagenicity observed in TA102 with S9mix, so additional mutagenic compounds might be produced by *A. fumigatus*.

To date, interactions between aflatoxins tested in mixtures have not been extensively investigated. Using the Ames test, only Said et al. [37] described a non-additive effect of AFB_1_ and N-acetylaminofluorene. Interaction mechanisms were more often studied towards various cell culture models or even in vivo. Hence, recently, Li et al. [31] reported a stronger renal toxicity of AFB_1_ and AFM_1_ when mixed, involving a pro-oxidant mechanism both in vitro (HEK 293 cells) and in vivo (CD-1 mice). Actually, AFB_1_ was most of the time tested in mixture with structurally distinct mycotoxins, such as fumonisin B_1_ (FB_1_) and ochratoxin (OTA). Antagonistic effect of (AFB_1_ + FB_1_) mixture was shown on HepG2 cells cytotoxicity whereas in contrast, synergistic mechanism was obtained on BEAS-2B cell as well as on F-344 rats mortality [38]. Theumer et al. [39] observed a more pronounced apoptosis in rat livers when FB_1_ and AFB_1_ were mixed, together with enhanced impairments in sphingolipid metabolism. They also reported significant DNA damage in spleen mononuclear cells from rats fed with this mixture [33]. On monkey kidney Vero cells, additive interactions were observed for (AFB_1_ + OTA) mix: cell viability was reduced and DNA damage was increased [40]. But in HCT-8 intestinal cancer cells, antagonistic effects were observed on various AFB_1_-induced biomarkers (DNA adducts formation, p53 induction or Mdm2 expression) [41]. Thus, no general rule could be inferred from these data, due to the variety of biological models, protocols, mycotoxin associations and mathematical models used to describe mycotoxin interactions. But the negative interaction observed in the present study for the reconstituted mixtures involving aflatoxins was in accordance with some previous experiments conducted on cell cultures.

## 4. Conclusions

After isolation of various fungal strains, we evaluated their toxigenic and mutagenic properties. We thus demonstrated that some of these isolates were able to produce mycotoxins in vitro which could result in a potential hazard for farmers as well as for livestock.

We also showed that, besides identified mycotoxins, additional secondary metabolites should contribute to the global mutagenic responses evaluated with the Ames test.

Among the identified aflatoxins, AFB_1_ was the sole mutagenic found when tested from pure standards in a range of doses corresponding to those measured in extracts. However, its association with AFB_2_ and AFM_1_ led to a decreased mutagenicity, indicating that AFB_2_ and/or AFM_1_ negatively modulate the toxicity of the corresponding mixture. AFB_1_ is rarely produced alone from fungi, and genotoxicity of combined mycotoxins is hard to predict. Thus, mutagenic activity assessment of the “natural” complex mixtures appears more informative and relevant than those of some isolated mycotoxins or reconstituted mixtures for hazard assessment.

Finally, the panel of *Salmonella* tester strains used indicates that various mechanisms were involved in the mutagenic responses. Besides the well documented formation of AFB_1_ 8,9-epoxide upon S9mix addition, and the subsequent formation of DNA adducts, a direct mutagenicity was also observed for higher doses in TA98 and TA100 which, therefore, did not involve exogenous activation. An additional contribution through oxidative damage was also detected upon addition of S9mix.

## 5. Materials and Methods

### 5.1. Strain Collection

The non toxigenic *A. flavus* strain was isolated from a bioaerosol sampled in a dairy cattle shed in Normandy [4]. The toxigenic *A. flavus* strain was purified from contaminated oilseed cakes, as well as the non toxigenic *A. parasiticus* strain [2]. The toxigenic *A. parasiticus* was obtained from a bioaerosol collected during the distribution of cattle feed [3].

The gliotoxin producer strain of *A. fumigatus* was isolated from a dairy cattle shed bioaerosol [4] whereas the non-producer strain was obtained from a contaminated oilseed cake sample [2].

All isolates were preserved on malt extract agar (MEA) in the laboratory mycological bank (stored at 4 °C).

### 5.2. Toxigenic Ability of Fungal Isolates

Each isolate was tested in triplicate for its ability to produce mycotoxins in vitro. Each strain was cultivated on MEA. The plates were incubated at 25 °C for two weeks and then three agar plugs measuring 8 mm in diameter were removed from the central area of the colony (including conidia and mycelium), pooled, weighted and transferred to 5-mL glass vials as previously described by Garon et al. [42]. Mycotoxins were extracted by 2 mL of ethyl acetate acidified with 1% acetic acid as recommended by Samson et al. [43]. After 15 min of centrifugation at 1500 rpm, each extract was evaporated to dryness under a stream of nitrogen. The residue was dissolved in 0.5 mL of an acetonitrile–water mixture (10:90 v/v) and filtered through Millex HV 0.45 µm before their injection into the high-performance liquid chromatography coupled with mass spectrometry (HPLC–MS/MS). Aflatoxins were then quantified by HPLC–MS/MS in multiple reaction monitoring (MRM) according to the method described by Pottier et al. [44]. Mycotoxins were quantified by HPLC–MS/MS in MRM. Liquid chromatography was performed using an Agilent Technologies 1200 HPLC system coupled to a triple quadrupole spectrometer (6460 series, Agilent Technologies, Santa Clara, CA, USA) equipped with an electrospray interface, operated in the positive and negative modes. The MassHunter B.02.00 software was used for data processing.

The analytes were chromatographed according to 2 chromatographic methods.

Eleven mycotoxins (aflatoxins B_1_, B_2_, G_1_, G_2_, M_1_, diacetoxyscirpenol, gliotoxin, mycophenolic acid, neosolaniol, ochratoxin A, T-2 toxin) were separated (Method 1) onto Zorbax SB, Rapid Resolution HT-C18 column (1.7 µm, 50 × 2 mm; Agilent Technologies) at 60 °C. Fumonisin B_1_-13C34 was used as internal standard. The injection volume of the samples on the analytical column was 10 µL. The mobile phase consisted of a variable mixture of acetonitrile (solvent A) and water added with formic acid 1% (solvent B) at a flow rate of 0.4 mL/min. A linear gradient was run starting with 10% to 100% solvent A over 10 min and staying at 100% over 1 min.

The mass spectrometer was operated in positive mode using dynamic MRM. The nebulizer gas and desolvatation gas were respectively nitrogen heated at 300 °C at 10 L/min and 400 °C at 12 L/min.

Nine mycotoxins (alternariol, deoxynivalenol, deepoxydeoxynivalenol, 3-acetyldeoxynivalenol, 15-acetyldeoxynivalenol, fusarenon X, HT-2 toxin, verrucarol, zearalenone) were separated (Method 2) onto a Zorbax Eclipse Plus, Rapid Resolution HD-C18 column (1.7 µm, 50 × 2 mm; Agilent Technologies) at 60 °C. Deoxynivalenol-13C15 was used as internal standard. The injection volume of the samples on the analytical column was 20 µL. The mobile phase consisted of a variable mixture of methanol (solvent A) and water (solvent B) at a flow rate of 0.4 mL/min. A linear gradient was run, starting with 10% to 100% solvent A over 10 min and staying at 100% over 1 min.

The mass spectrometer was operated in both negative and positive modes using MRM. Three retention windows were defined according to the retention time and the optimized ESI mode. The nebulizer gas and desolvatation gas were nitrogen heated at 250 °C at 10 L/min (excepted for the third retention window, at 12 L/min) and 400 °C at 12 L/min respectively.

Other common parameters used for the mass spectrometer were as follow: capillary voltage, 4.0 kV; pressure of nebulization, 45 psi; nozzle voltage, 300 V.

The most abundant product ion after collision-induced fragmentation was used for quantitative purposes, and the second product ion for confirmation. The linearity was done by spiking increasing concentrations (triplicate) of the mycotoxin standards (0.1 to 50 µg/L). The quantification and detection limits (QL and DL) were determined by spiked samples based on signal to noise ratio of 10:1 for quantification, and 3:1 for detection limit.

Using this multimycotoxin protocol, 6 mycotoxins were found from fungal cultures, namely aflatoxin B_1_, B_2_, and M_1_, fumagillin, gliotoxin and verruculogen. The quantification limits were 0.5 µg/L for AFB_1_, AFB_2_, AFM_1_, fumagillin and verruculogen, and 2.5 µg/L for gliotoxin.

### 5.3. Mutagenicity

#### 5.3.1. Extract Preparation

For the mutagenic potential evaluation, the dry residues obtained above were dissolved in 550 µL DiMethylSulfOxyde (DMSO) and filtered through Millex PTFE HV 0.45 μm (i.e; resistant Teflon).

These extracts were abbreviated as follow: (Flav^+/−^) = extracts obtained from *A. flavus* isolates (^+/−^ for toxigenic/not toxigenic strain), (Para^+/−^) = extracts obtained from *A. parasiticus* isolates and (Fum_Glio+/−_) = extracts obtained from *A. fumigatus* (both strains synthesized fumagillin and verruculogen, but _Glio+/−_ distinguish strains that are able or not to synthesize gliotoxin).

#### 5.3.2. Mixture of Mycotoxins

Standard mycotoxins aflatoxin B_1_, B_2_, M_1_ (certified grade, Cluzeau Info Labo, Ste Foy la Grande, France); fumagillin, gliotoxin, and verruculogen (Sigma-Aldrich (St. Louis, MO, USA) were tested alone and mixed at identical concentrations to those found in extracts.

#### 5.3.3. Ames Test Procedure

Overnight cultures (12 h at 37 °C with continuous shaking) of *Salmonella typhimurium* tester strains TA98, TA100 and TA102 (Trinova Biochem, Giessen, Germany) were obtained by addition of 30µL of frozen culture in 10 mL of Oxoid nutrient broth N°2 solution (0.25 g/10 mL distillated water). The Ames test included a preincubation step as previously described [44]. Briefly, 10 µl of samples (extracts, pure mycotoxins or their mix dissolved in DMSO) were mixed with 100 µl of bacterial overnight culture and either 100 µL of S9mix (prepared with 5% S9) or 100 µL phosphate buffer (for conditions without S9mix). The S9 fraction was obtained from livers of Aroclor 1254-induced Sprague–Dawley male rats (Moltox, Boone, NC, USA). After 60 min shaking (185 rpm at 37 °C), 2 mL of molten agar (agar 0.6% and NaCl 0.5% *w*/*v* in distillated water) supplemented with histidine- and biotin traces (final concentration 0.5 g/L and 0.012 g/L for histidine and biotin respectively) were added to the tubes and quickly poured onto minimal glucose plates (containing agar, glucose and mineral salts; for detailed composition, see Maron and Ames [45]). After 48 h incubation at 37 °C, the number of revertants was automatically counted (Noesis software, Saint Aubin, France). For each sample, three concentrations were tested, in triplicate. Results were repeated in two to four independent experiments. Previous studies led us to consider a significant mutagenicity when the ratio (Induced revertants/Spontaneous revertants) was >2 for TA98, ratio > 1.6 for TA100 and ratio > 1.3 for TA102, together with a dose-effect relationship [46]. Toxicity was evaluated in parallel by the microscopic observation of the background lawn density.

Strain-specific positive controls were used for each experiment: 2-nitrofluorene 2.5 μg/plate (TA98 wo S9mix: 886 revertants ± 161), 2-aminofluorene 0.5 µg/plate (TA98 with S9mix: 704 revertants ± 87), sodium azide 1.5 μg/plate (TA100 wo S9mix: 602 revertants ± 91) and ter-butylhydroperoxide 2 µmol/plate (TA102 wo S9Mix: 1377 revertants ± 148).

### 5.4. Statistical Analysis

An analysis of covariance (ANCOVA) was performed with all significant mutagenic data.

Three kinds of sample were tested, namely fungal extracts, mycotoxin mix and pure mycotoxins. A *p* value < 0.05 was considered significant. When the difference between these samples was significant, an additional comparison by Student’s test using a Bonferroni adjustment was performed.

## Figures and Tables

**Table 1:** Mycotoxigenicity of the extracts prepared from various *Aspergillus* strains.

Extracts	Mycotoxins (µg/g Plug)		
	AFB_1_	AFB_2_	AFM_1_
(Flav +)	12.14	0.23	0.20
(Flav −)	<QL	<QL	<QL
(Para +)	0.30	0.006	<QL
(Para −)	<QL	<QL	<QL
	**GLIO**	**VER**	**FUM**
(Fumi_Glio+_)	11.6	0.04	0.23
(Fumi_Glio−_)	<QL	0.07	0.13

AFB_1_: aflatoxin B_1_, AFB_2_: aflatoxin B_2_, AFM_1_: aflatoxin M_1,_ GLIO: gliotoxin, VER: verruculogen, FUM: fumagillin; +: mycotoxigenic strain; **−**: non mycotoxigenic strain; _Glio+/__−_: strain producing/not producing gliotoxin; <QL: below to the limit of quantification (2.5 ng/g for gliotoxin and 0.5 ng/g for other mycotoxins).

**Table 2:** Comparative mutagenicity of *Aspergillus flavus* (Flav^+/^^−^) extracts versus equivalent mix of mycotoxins, evaluated in *Salmonella* tester strains TA98, TA100 and TA102 without (−S9) metabolic activation.

Extract Type	Mycotoxins (µg/Plate)			Mutagenicity (−S9 mix) (Rev/Plate ^a^)					
	AFB_1_	AFB_2_	AFM_1_		Whole Extract	AFB_1_+AFB_2_	AFB_1_+AFM_1_	AFB_2_+AFM_1_	AFB_1_+AFB_2_+AFM_1_
**(Flav−)**	<QL	<QL	<QL	**TA98–S9** SRN = 26 ± 10	17 ± 4	-	-	-	
(Flav+)	0.7500	0.0140	0.0130		**362 ± 25**	**73 ± 7**	**61 ± 7**	12 ± 5	**76 ± 9**
	0.3000	0.0056	0.0052		**106 ± 6**	**45 ± 6**	**45 ± 5**	16 ± 9	**48 ± 10**
	0.1500	0.0028	0.0026		**41 ± 8**	33 ± 7	**42 ± 4**	18 ± 12	**41 ± 15**
(Flav−)	<QL	<QL	<QL	**TA100–S9** SRN = 146 ± 32	105 ± 12	-	-	-	-
(Flav+)	0.5000	0.0093	0.0087		**1031 ± 178**	**240 ± 33**	**231 ± 56**	127 ± 14	184 ± 23
	0.3000	0.0056	0.0052		**685 ± 28**	**222 ± 21**	181 ± 49	144 ± 13	205 ± 39
	0.1500	0.0028	0.0026		**344 ± 35**	190 ± 31	154 ± 29	151 ± 23	140 ± 23
(Flav−)	<QL	<QL	<QL	**TA102–S9** SRN = 485 ± 63	427 ± 40	-	-	-	-
(Flav+)	0.7500	0.0140	0.0130		446 ± 10	507 ± 37	492 ± 61	490 ± 57	509 ± 32
	0.5000	0.0093	0.0087		475 ± 41	473 ± 39	471 ± 46	462 ± 73	458 ± 108
	0.1500	0.0028	0.0026		490 ± 25	511 ± 49	478 ± 55	444 ± 63	502 ± 37

AFB_1_: aflatoxin B_1_, AFB_2_: aflatoxin B_2_, AFM_1_: aflatoxin M_1;_ (Flav−): extract prepared from non toxigenic *A. flavus;* ^a^ mean revertant number ± standard deviation (SD); (Flav+): extract prepared from toxigenic *A. flavus* SRN: spontaneous revertant number (mean ± SD); <QL: below to the limit of quantification; **In bold:** significant mutagenicity.

**Table 3:** Comparative mutagenicity of *Aspergillus flavus* (Flav^+/-^) extracts versus equivalent mix of mycotoxins, evaluated in *Salmonella* tester strains TA98, TA100 and TA102 with (+S9) metabolic activation.

Extract Type	Mycotoxins (µg/Plate)			Mutagenicity (+S9mix) (Rev/Plate ^a^)					
	AFB_1_	AFB_2_	AFM_1_		Whole Extract	AFB_1_+AFB_2_	AFB_1_+AFM_1_	AFB_2_+AFM_1_	AFB_1_+AFB_2_+AFM_1_
**(Flav−)**	<QL	<QL	<QL	**TA98 + S9** SRN = 27 ± 10	28 ± 6	-	-	-	-
(Flav+)	0.0750	0.0014	0.0013		**2154 ± 111**	**603 ± 83**	**413 ± 71**	20 ± 13	**200** ± 38
	0.0250	0.0005	0.0004		**1183 ± 61**	**248 ± 72**	**123 ± 14**	22 ± 8	50 ± 3
	0.0050	0.0001	0.0001		**183 ± 26**	53 ± 8	47 ± 5	21 ± 11	63 ± 34
(Flav−)	<QL	<QL	<QL	**TA100 + S9** SRN = 156 ± 43	105 ± 25	-	-	-	-
(Flav+)	0.0250	0.0005	0.0004		**1905 ± 39**	**635 ± 56**	**783 ± 55**	153 ± 39	**306 ± 51**
	0.0050	0.0001	0.0001		**861 ± 84**	**253 ± 23**	**265 ± 12**	152 ± 14	136 ± 61
	0.0010	0.00002	0.00002		227 ± 36	nd	nd	nd	185 ± 22
(Flav−)	<QL	<QL	<QL	**TA102 + S9** SRN = 480 ± 65	409 ± 11	-	-	-	-
(Flav+)	0.5000	0.0100	0.0080		**2222 ± 75**	539 ± 67	467 ± 78	481 ± 71	500 ± 26
	0.3000	0.0030	0.0048		**1699 ± 69**	529 ± 56	497 ± 40	475 ± 87	493 ± 23
	0.1500	0.0015	0.0024		**806 ± 41**	578 ± 130	458 ± 29	488 ± 132	502 ± 67

AFB_1_: aflatoxin B_1_, AFB_2_: aflatoxin B_2_, AFM_1_: aflatoxin M_1;_ (Flav−): extract prepared from non toxigenic *A. flavus;* ^a^ mean revertant number ± SD; (Flav+): extract prepared from toxigenic *A. flavus* SRN: spontaneous revertant number (mean ± SD); nd: not determined; <QL: below to the limit of quantification; **In bold:** significant mutagenicity.

**Table 4:** Comparative mutagenicity of *Aspergillus parasiticus* (Para^+/^^−^) extracts versus equivalent mix of mycotoxins, evaluated in *Salmonella* tester strains TA98, TA100 and TA102 without (−S9) metabolic activation.

Extract Type	Mycotoxins (µg/Plate)		Mutagenicity (−S9mix) (Rev/Plate ^a^)		
	AFB_1_	AFB_2_		Whole Extract	AFB_1_+AFB_2_
(Para−)	<QL	<QL	**TA98 – S9** SRN = 26 ± 10	27 ± 4	-
(Para+)	0.3000	0.0050		**249 ± 17**	**76 ± 9**
	0.1500	0.0025		**213 ± 16**	48 ± 10
	0.0500	0.0008		**68 ± 20**	41 ± 15
(Para−)	<QL	<QL	**TA100 – S9** SRN = 146 ± 32	116 ± 7	-
(Para+)	0.3000	0.0050		149 ± 19	184 ± 23
	0.1500	0.0025		167 ± 24	205 ± 39
	0.0500	0.0008		139 ± 4	140 ± 23
(Para−)	<QL	<QL	**TA102 – S9** SRN = 485 ± 63	372 ± 22	-
(Para+)	0.3000	0.0050		470 ± 22	509 ± 32
	0.1500	0.0025		475 ± 19	458 ± 108
	0.0500	0.0008		450 ± 26	502 ± 37

(Para−): extract from non toxigenic *A. parasiticus*; (Para+): extract from toxigenic *A. parasiticus.*

**Table 5:** Comparative mutagenicity of *Aspergillus parasiticus* (Para^+/-^) extracts versus equivalent mix of mycotoxins, evaluated in *Salmonella* tester strains TA98, TA100 and TA102 with (+S9) metabolic activation.

Extract Type	Mycotoxins (µg/Plate)		Mutagenicity (+S9mix) (Rev/Plate ^a^)		
	AFB_1_	AFB_2_		Whole Extract	AFB_1_+AFB_2_
(Para−)	<QL	<QL	**TA98 + S9** SRN = 26 ± 10	23 ± 1	-
(Para+)	0.0050	0.0001		**358 ± 36**	**125 ± 40**
	0.0025	0.00005		**141 ± 19**	**57 ± 8**
	0.0010	0.00002		**60 ± 1**	29 ± 2
(Para−)	<QL	<QL	**TA100 + S9** SRN = 146 ± 32	126 ± 15	-
(Para+)	0.0050	0.0001		**487 ± 27**	**232 ± 8**
	0.0025	0.00005		**247 ± 14**	187 ± 8
	0.0010	0.00002		160 ± 19	142 ± 27
(Para−)	<QL	<QL	**TA102 + S9** SRN =4 80 ± 65	446 ± 20	-
(Para+)	0.3000	0.0050		**751 ± 38**	**759 ± 36**
	0.1500	0.0025		664 ± 63	579 ± 21
	0.0500	0.0008		528 ± 4	489 ± 23

AFB_1_: aflatoxin B_1;_ AFB_2_: aflatoxin B_2_*;* (Para−): extract from non toxigenic *A. parasiticus*; (Para+): extract from toxigenic *A. parasiticus;* ^a^ mean revertant number; SRN: spontaneous revertant number (mean ± SD)*;* <QL: below to the limit of quantification; **In bold**: significant mutagenicity.

**Table 6:** Comparative mutagenicity of toxigenic *Aspergillus fumigatus* (Fumi_Glio+/−_) extracts versus equivalent mix of pure mycotoxins, evaluated in *Salmonella* tester strains TA98, TA100 and TA102 without (−S9) metabolic activation.

Extract Type	[Mycotoxins] (µg/Plate)				Mutagenicity (−S9mix) (Rev/Plate ^a^)						
	GLIO	FUM		VER		Whole Extract			FUM+VER ± GLIO		
(Fumi_Glio+_)	2.050	0.040	0.010		**TA98 – S9** SRN = 20 ± 3	22	±	8	22	±	7
	0.410	0.008	0.002			18	±	4	23	±	2
(Fumi_Glio−_)	<QL	0.0130	0.0240			17	±	4	26	±	7
	<QL	0.0026	0.0048			19	±	1	24	±	1
(Fumi_Glio+_)	2.050	0.040	0.010		**TA100 – S9** SRN=122 ± 22	179	±	44	141	±	6
	0.410	0.008	0.002			142	±	6	141	±	7
(Fumi_Glio−_)	<QL	0.0130	0.0240			123	±	9	119	±	5
	<QL	0.0026	0.0048			147	±	17	125	±	25
(Fumi_Glio+_)	2.050	0.040	0.010		**TA102 – S9** SRN = 424 ± 13	494	±	137	432	±	48
	0.410	0.008	0.002			443	±	37	423	±	45
(Fumi_Glio−_)	<QL	0.0130	0.0240			462	±	23	403	±	40
	<QL	0.0026	0.0048			395	±	16	417	±	15

FUM: fumagillin; GLIO: gliotoxin; VER: verruculogen; (Fumi_Glio+_): extract from *A. fumigatus* gliotoxin producer; (Fumi_Glio-_): extract from *A. fumigatus* gliotoxin not producer; ^a^ mean of the revertant number; SRN: spontaneous revertant number (mean ± SD); <QL: below to the limit of quantification.

**Table 7:** Comparative mutagenicity of toxigenic *Aspergillus fumigatus* (Fumi_Glio+/-_) extracts versus equivalent mix of pure mycotoxins, evaluated in *Salmonella* tester strains TA98, TA100 and TA102 with metabolic activation (+S9).

Extract Type	[Mycotoxins] (µg/Plate)			Mutagenicity (Rev/Plate ^a^)						
	GLIO	FUM	VER		Whole Extract			FUM+VER±GLIO		
(Fumi_Glio+_)	2.050	0.040	0.010	**TA98 + S9** SRN = 19 ± 2	23	±	1	22	±	1
	0.410	0.008	0.002		21	±	7	22	±	2
(Fumi_Glio−_)	<QL	0.0130	0.0240		18	±	1	22	±	1
	<QL	0.0026	0.0048		16	±	5	22	±	2
(Fumi_Glio+_)	2,050	0.040	0.010	**TA100 + S9** SRN = 92 ± 23	103	±	9	116	±	18
	0,410	0.008	0.002		73	±	5	122	±	9
(Fumi_Glio−_)	<QL	0.0130	0.0240		123	±	21	117	±	10
	<QL	0.0026	0.0048		95	±	16	130	±	8
(Fumi_Glio+_)	2.050	0.040	0.010	**TA102 + S9** SRN = 388 ± 28	**539**	±	**37**	373	±	29
	0.410	0.008	0.002		442	±	3	395	±	24
(Fumi_Glio−_)	<QL	0.0130	0.0240		**516**	±	**34**	393	±	11
	<QL	0.0026	0.0048		392	±	22	361	±	54

FUM: fumagillin; GLIO: gliotoxin; VER: verruculogen; (Fumi_Glio+_): extract from *A. fumigatus* gliotoxin producer; (Fumi_Glio-_): extract from *A. fumigatus* gliotoxin not producer; ^a^ mean of the revertant number; SRN: spontaneous revertant number (mean ± SD); <QL: below to the limit of quantification; **In** **bold**: samples displaying a significant mutagenicity.

**Table 8:** Mutagenicity of standard mycotoxins evaluated in *Salmonella* tester strains TA98, TA100 and TA102 without (−S9) or with (+S9) metabolic activation.

Mycotoxins		Concentration (µg/Plate)	Mutagenicity (Revertants/Plate ^a^)		
			TA98	TA100	TA102
**None**	**−S9 +S9**	0 0	26 ± 10 27 ± 10	146 ± 32 156 ± 43	485 ± 63 480 ± 65
**AFB_1_**	−S9	0.7500 0.5000 0.3000 0.1500	**106 ± 40** nd **53 ± 14** 29 ± 10	nd **272 ± 95** 201 ± 48 154 ± 35	419 ± 86 458 ± 73 nd 472 ± 91
	+S9	0,5000 0.3000 0.1500 0.0750 0.0250 0.0050 0.0010	nd nd (T) **1075 ± 112** **275 ± 20** **88 ± 13** nd	nd nd nd (T) **1050 ± 161** **279 ± 37** 139 ± 38	473 ± 112 482 ± 133 501 ± 104 nd nd nd nd
**AFB_2_**	−S9	0.0140 0.0050 0.0010	20 ± 3 20 ± 2 20 ± 2	132 ± 7 128 ± 6 127 ± 17	461 ± 14 476 ± 27 449 ± 26
	+S9	0.0140 0.0050 0.0010	28 ± 4 25 ± 6 23 ± 1	142 ± 12 157 ± 9 127 ± 2	416 ± 18 418 ± 22 429 ± 17
**AFM_1_**	−S9	0.0130 0.0020	18 ± 2 18 ± 3	186 ± 2 183 ± 10	461 ± 14 463 ± 45
	+S9	0.0013 0.0002	23 ± 4 32 ± 2	161 ± 11 168 ± 6	475 ± 45 439 ± 27
**FUM**	−S9	0.0400 0.0250 0.0090	18 ± 2 19 ± 2 22 ± 1	140 ± 10 149 ± 6 148 ± 9	468 ± 34 451 ± 38 439 ± 6
	+S9	0.0400 0.0250 0.0090	30 ± 11 28 ± 2 31 ± 1	133 ± 3 123 ± 19 127 ± 2	460 ± 7 485 ± 30 493 ± 17
**GLIO**	−S9	2.0500 0.4100	17 ± 4 20 ± 4	130 ± 8 131 ± 1	457 ± 18 454 ± 20
	+S9	2.0500 0.4100	20 ± 1 22 ± 1	148 ± 8 148 ± 7	414 ± 17 388 ± 21
**VER**	−S9	0.0100 0.0050	17 ± 2 19 ± 1	141 ± 12 145 ± 10	442 ± 30 467 ± 19
	+S9	0.0100 0.0050	28 ± 3 28 ± 5	157 ± 6 158 ± 13	494 ± 8 349 ± 12

AFB_1_: aflatoxin B_1_, AFB_2_: aflatoxin B_2,_ AFM_1_: aflatoxin M_1_, FUM: fumagillin, GLIO: gliotoxin, VER: verruculogen; nd: not determined; (T) toxicity (lower density of the microscopic background lawn); ^a^ mean of the revertant number; In **bold**: samples displaying a significant mutagenicity.

**Table 9:** Comparative mutagenic potential of toxigenic *A. flavus* and *A. parasiticus* extracts versus pure AFB_1_ in *Salmonella* tester strains TA98, TA100 and TA102 without (−) or with (+) metabolic activation (S9).

Strains	S9	Revertants/ng_AFB1_		
		Extracts		Pure AFB_1_
		(Flav^+^)	(Para^+^)	
**TA98**	−	0.5	1.4	0.18
	+	47.3	71.6	17.6
**TA100**	−	2.3	-	0.5
	+	172.2	99	55.8
**TA102**	−	-	-	-
	+	5.7	4.4	-

-: not mutagenic.
